# Water mites (Acari, Hydrachnidia) from Baishih River drainage in Northern Taiwan, with description of two new species

**DOI:** 10.3897/zookeys.203.3356

**Published:** 2012-06-20

**Authors:** Vladimir Pešić, Rita S.W. Yam, Benny K. K. Chan, Tapas Chatterjee

**Affiliations:** 1 Department of Biology, University of Montenegro, Cetinjski put b.b., 81000 Podgorica, Serbia and Montenegro; 2 Department of Bioenvironmental Systems Engineering, National Taiwan University, Taipei 10617, Taiwan R.O.C.; 3 Biodiversity Research Center, Academia Sinica, Taipei 115, Taiwan R.O.C.; 4 Department of Biology, Indian School of Learning, I.S.M. Annexe, P.O. – I.S.M., Dhanbad-826004, Jharkhand, India

**Keywords:** Acari, water mites, new species, running waters, Baishih River drainage, Taiwan

## Abstract

New records of water mites (Acari: Hydrachnidia) from Baishih River drainage of north Taiwan, are presented. Twelve species are recorded, of which ten are new for Taiwan; two of them, *Torrenticola projectura* and *Hygrobates taiwanicus* are described as new for science.

## Introduction

Taiwan is a large island (35, 881 km^2^) located in the Asian Pacific region. As the Tropic of Cancer bisects the Island, the climate changes from subtropical to tropical from the northern to southern parts of the island. The climate of Taiwan is heavily influenced by monsoons and typhoons which bring an annual rainfall of ~ 2150 mm, with 80% of precipitation concentrated in the summer (or wet season), i.e. May–October. As there is a central mountain range extending from the north to the south of the island with surrounding plateau and hills, two third of the island is covered by mountainous area with an elevation 100–>3000 m. All rivers in Taiwan are characterized by short length and steep gradient with rapid flows and small drainage basins associated with the steep terrain. Such diverse patterns of geographical typology and hydrology result in the high levels of habitat heterogeneity and aquatic biodiversity of the Taiwan rivers (Shih et al. 2007).

**Figure 1.**
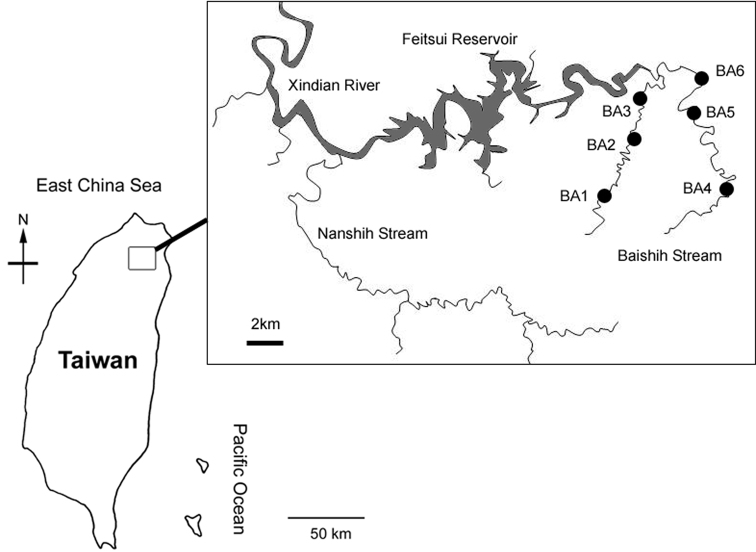
Map of study area showing location of the six sampling sites.

Water mites are one of the most ubiquitous components of the lotic communities. However, detailed investigation on their taxonomy, distribution and ecology is generally lacking in the Asian Pacific region. Furthermore, the water mite fauna of Taiwan is very incompletely known. Recently, we published the results of the first collection of water mites from Taiwan, listing three species of the family Torrenticolidae, two of them were new to science (Pešić et al. 2011). During 2009–2010, in our study of the ecological responses of aquatic macrozoobenthos to land-use and environmental disturbances in Baishih River from the northern Taiwan (Fig. 1), we conducted a bi-monthly biodiversity survey at the six study sites located in this drainage network. The present samples of water mites were collected in this survey.

Baishih River system is located in the northern Taiwan. It is one of the major upstream feeder tributary of the urbanised Xindian River which runs into Danshui River in the New Taipei City. Baishih River originates from both Mount Sanfonsun and Mount Yingtzuling, and drains southwestward into Feitsui Reservoir which provides water supply for the population of the Taipei metropolitan area. Baishih River is ~50 km long and the catchment area is about 310 km^2^. The present study focused on two major tributaries Chinkualiao Stream and Diyu Stream of Baishih River drainage. All study sites are relatively undisturbed by human activities as both Chinkualiao Stream and Diyu Stream are located within the protected area. The riparian area is dominated by natural forest with discontinuous distribution of cultivated land owned by local villagers.

The paper aims to describe the diversity and distribution pattern of water mites from the northern Taiwan. In the present study, twelve species are identified, two of them are new to science. Descriptions of these species are given in this paper.

## Material and methods

**Figure 2.**
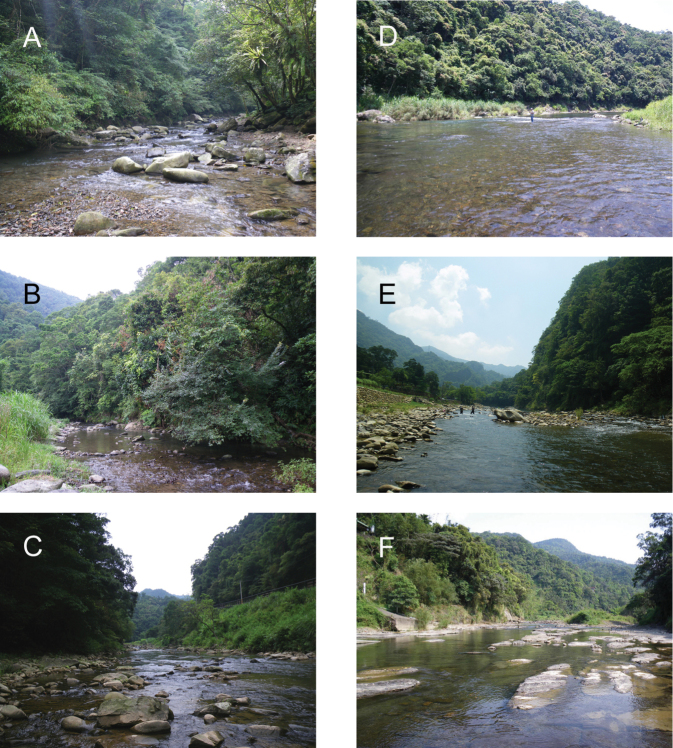
Photographs of the six study sampling sites in the present study. **A** = BA-1 **B** = BA-2 **C** = BA-3 **D** = BA-4 **E** = BA-5 **F** = BA-6.

In this study, water mites were collected at six sampling sites (Table 1; Figs 1, 2A–F) using standard Surber sampling method with WaterMark^®^ Surber Type Stream Bottom Sampler (500 μm mesh). Water mites were sorted in the laboratory with the aid of a stereo microscope and preserved in 90% ethanol. All the material has been collected by Rita Yam and this is not repeated in the text. Holotypes and paratypes of the new species are deposited in the National Museum of Natural Science (NMNS), Taichung, Taiwan. Other materials are kept in the collections of the Ecology and Conservation Laboratory, Department of Bioenvironmental Systems Engineering, National Taiwan University (ECL).

**Table 1.** List of the sampling sites in the present study.

**Site code**	**Latitude**	**Longitude**	**Catchment**	**Subcatchment**	**Altitude (m asl)**
BA-1	121.656242°E, 24.882695°N		Baishih River	Chinkualiao Stream	365
BA-2	121.656328°E, 24.883970°N		Baishih River	Chinkualiao Stream	343
BA-3	121.674029°E, 24.924912°N		Baishih River	Chinkualiao Stream	250
BA-4	121.701640°E, 24.908065°N		Baishih River	Diyu Stream	232
BA-5	121.697561°E, 24.915911°N		Baishih River	Diyu Stream	210
BA-6	121.696603°E, 24.933962°N		Baishih River	Diyu Stream	190

In the section ‘Material examined’, the sampling site codes were derived from the geographical database Rita Yam (see Table 1). The composition of the material is given as: males/females/deutonymphs or adults/deutonymphs. All measurements are given in µm. The following abbreviations are used: asl = above sea level, Cx-I = first coxae, Cxgl-4 = coxoglandularia of fourth coxae, dL = dorsal length, H = height, L = length, W = width, I-L-6 = Leg 1, sixth segment (tarsus), mL = medial length, n = number of specimens examined, P-1 = palp, first segment, Vgl-2 = ventroglandulare 2.

## Systematics

### Family Hydrodromidae K. Viets. Genus *Hydrodroma* Koch, 1837

#### Hydrodroma cf. rheophila

Cook, 1967

http://species-id.net/wiki/Hydrodroma_rheophila

Fig. 3A

##### Material examined.

ECL-BA-1: 01.vii.2010 0/1/0 (mounted).

##### Remarks.

**Figure 3.**
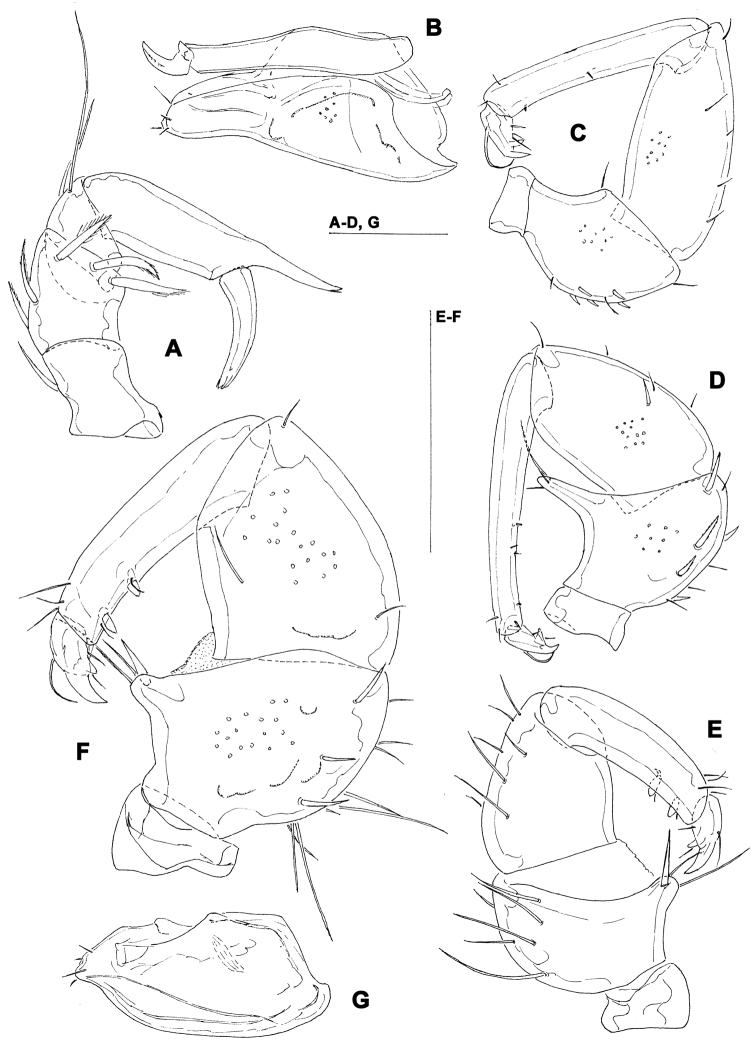
**A** *Hydrodroma cf. rheophila* Cook, 1967, female: palp. **B–C** *Sperchon rostratus* Lundblad, 1969, female: **B** = palp **C** = capitulum and chelicera **D** *Sperchon cf. gracilipalpis* Lundblad, 1941, female: palp **E–G** *Sperchon cornutoides* Lundblad, 1941 (**F–G** = female, **E** = male) **E–F** = palp **G** = capitulum. Scale bars = 100 µm.

Due to the presence of a single, short swimming hair on segments four and five of the third and fourth legs and the shape of palp (Fig. 3A), the single specimen from the Chinkualiao stream shows general conformity with *Hydrodroma rheophila*. As Pešić et al. (2010) noted the variability of additional populations tentatively assigned to *Hydrodroma rheophila* (e.g. from Greece and Oman, see Pešić et al. 2010 and Smit and Pešić 2010, respectively) from different regions of the whole distribution area needs to be examined to clarify the taxonomy. This probably will require the use of molecular approach to examine the degree of genetic differentiation among the geographical populations.

##### Distribution.

India, Indonesia, Iran, Oman, Balkan. New for Taiwan.

### Family Sperchontidae Thor. Genus *Sperchon* Kramer, 1877

#### Sperchon (Hispidosperchon) rostratus

Lundblad, 1969

http://species-id.net/wiki/Sperchon_rostratus

Figs 3B–C

##### Material examined.

ECL-BA-3: iii.2010 0/1/0 (mounted). ECL-BA-5: iii.2010 0/1/0.

##### Remarks.

The two females examined in the present study fit well the description of *Sperchon rostratus* Lundblad, 1969. Figures 3B–C show some morphological details of the specimen from Chinkualiao stream.

##### Distribution.

Burma, China (Guizhou Province), Turkey, Iran. New forTaiwan.

#### Sperchon (Hispidosperchon) cf. gracilipalpis

Lundblad, 1941

http://species-id.net/wiki/Sperchon_gracilipalpis

Fig. 3D

##### Material examined.

ECL-BA-2: iii.2010 1/1/0. ECL-BA-3: vi.2010 0/1/0 (mounted). ECL-BA-4: iii.2010 5/0 1/0/0 (mounted); iv.2010 0/1/0; 27.vii.2010 1/1/0. ECL-BA-5: iii.2010 1/1/0; iv.2010 0/1/0. ECL-BA-6: iv.2010 0/2/0.

##### Remarks.

The specimens from Baishih River drainage are provisionally assigned to the Oriental species *Sperchon gracilipalpis* Lundblad, 1941. However, they resemble both *Sperchon gracilipalpis* and the Palaearctic *Sperchon hispidus* Koenike, 1895 (common character state: dorsum of both sexes with seven paired and one unpaired (occasionally paired) muscle attachment plates; III/IV-L-3-5 with numerous pinnate dorsal setae; similar shape of palp (Fig. 3D) and male ejaculatory complex). The diagnostic differences separating these two species have never been discussed. More material of *Sperchon gracilipalpis* from the type area should be investigated in order to get an insight on further diagnostic differences.

##### Distribution.

SE Asia, China (Guizhou Province). New forTaiwan.

#### Sperchon cornutoides

Lundblad, 1941

http://species-id.net/wiki/Sperchon_cornutoides

Figs 3E–G

##### Material examined.

ECL-BA-1: 10.viii.2009 0/1/0; iii.2010 0/1/0; 01.vii.2010 0/1/0 (mounted); 02.vii.2010 0/2/0; 30.x.2010 1/0/0. ECL-BA-2: 27.vii.2010 2/0/0 (1/0/0 mounted). ECL-BA-3: 23.ix.2009 0/1/0; iii.2010 0/1/0. ECL-BA-4: iii.2010 0/1/0; 27.vii.2010 1/2/0 (1/1/0 mounted). ECL-BA-5: iv.2010 0/1/0. ECL-BA-6: iii.2010 0/1/0.

##### Remarks.

The specimens examined from Baishih River drainage agrees with the description of *Sperchon cornutoides* Lundblad, 1941, a species known only from Java (Lundblad 1936, 1971). This species closely resembles to *Sperchon cornutus* K. Viets, 1935, which can be easily distinguished by the claws bearing two clawlets instead of a claw with one clawlet in *Sperchon cornutoides*. *Sperchon xiaoqikongensis* Zhang & Jin, 2012, a species recently described from mainland China (Guizhou Province, Zhang et al. 2012) resemble the both aforementioned species due to shape of palp and capitulum. According to the original description (Zhang et al. 2012) this species differs from *Sperchon cornutoides* in the absence of muscle attachment plates, shorter peg-like seta on P-2 ventral projection and the presence of peg-like setae on P-2 and P-3 (as shown by figure of Zhang et al. 2012, Fig. 18). It is possible, especially in the case of weakly sclerotized specimens or due the clearing treatment with lactic acid, that muscle attachment plates could easily have been unobserved. The taxonomic state and relationships of this species should be investigated with further specimens from the type area.

##### Distribution.

Indonesia. New for Taiwan.

### Family Torrenticolidae Piersig. Genus *Monatractides* K. Viets, 1926

#### Monatractides cf. circuloides

(Halík, 1930)

http://species-id.net/wiki/Monatractides_circuloides

##### Material examined.

ECL-BA-1: 07.vii.2009 1/1/0; 23.ix.2009 2/0; iv.2010 13/0; 01.vi.2010 1/0; vii.2010 29/0; 27.vii.2010 2/1/0. ECL-BA-2: 02.ix.2009 11/2(1juvenile)/0; ii.2010 57/0; iii.2010 3/1/1; iv.2010 0/2/0; vi.2010 2/2/0; 27.vii.2010 17/0; 29.ix.2010 5/0; 30.x.2010 1/0. ECL-BA-3: 01.vii.2009 1/0/0; 23.vii.2009 1/0/0; ii.2010 4/11/0; ii.2010 10/0; iv.2010 0/1/0; 27.vii.2010 1/0/0. ECL-BA-4: 02.vii.2009 1/0/0; ii.2010 2/1/0; iii.2010 6/4/0; iv.2010 0/1/0; vi.2010 1/0/0; vii.2010 19/0; 27.vii.2010 5/0/0; 30.x.2010 1/0. ECL-BA-5: iii.2010 1/1/0; 01.vi.2010 4/0/0; vii.2010 11/0; 07.vii.2010 1/0/0; 30.x.2010 1/0/0. ECL-BA-6: 17.vi.2009 3/0; iii.2010 2/9/0; vi.2010 0/1/0; 01.vii.2010 1/0/0; 27.vii.2010 1/0/0; 29.ix.2010 1/0.

##### Remarks.

It is the most abundant species in our material. For an analysis of diagnostic characters of populations from Taiwan tentatively assigned to *Monatractides circuloides* see: Pešić et al. (2011).

##### Distribution.

Malaysia, Thailand, Taiwan.

### Genus *Torrenticola* Persig, 1896

#### Torrenticola taiwanicus

Pešić, Semenchenko, Chatterjee, Yam & Chan, 2011

http://species-id.net/wiki/Torrenticola_taiwanicus

##### Material examined.

ECL-BA-1: 07.vii.2009 1/0/0. ECL-BA-3: 27.vii.2010 1/4/0. ECL-BA-4: 02.vii.2009 0/2/0; ii.2010 0/1/0; iii. 2010 0/4/0; iv.2010 0/1/0 (mounted); vi.2010 1/1/0; 27.vii.2010 0/1/0; ECL-BA-5: 21.vii.2009 1/0/0; iii.2010 0/2/0; 01.vi.2010 1/1/0; 07.vii.2010 0/1/0. ECL-BA-6: ii.2010 1/0/0; iii. 2010 0/6/0; 27.vii.2010 0/1/0; 29.ix.2010 3/0.

##### Remarks.

The studied material was collected from February to September. For an analysis of diagnostic characters of *Torrenticola taiwanicus* see: Pešić et al. (2011).

##### Distribution.

Taiwan.

#### Torrenticola projectura sp. n.

urn:lsid:zoobank.org:act:41E5931A-EFE2-42B6-B0D5-A86EB897B9FF

http://species-id.net/wiki/Torrenticola_projectura

Figs 4A–E

##### Type series.

Holotype male (NMNS-6894-002), dissected and slide mounted, Taiwan, BA-1, Baishih River drainage, Chinkualiao Stream, 27.vii.2010. Paratype: one male, same data as holotype; one male, dissected and slide mounted, BA-2, Chinkualiao Stream, 02.ix.2009.

##### Diagnosis.

Male (female unknown). Cx-IV extending posterior to genital field; excretory pore posterior to line of primary sclerotization, Vgl-2 slightly posterior to excretory pore; capitulum with a short rostrum; medial suture line of Cx-II+III short; P-2 ventrodistal projection cone-shaped, pointed towards distal, P-3 ventrodistal projection larger than projection of P-2, P-3 with a long tapering ventral protrusion which curves distally.

##### Description.

**Figure 4.**
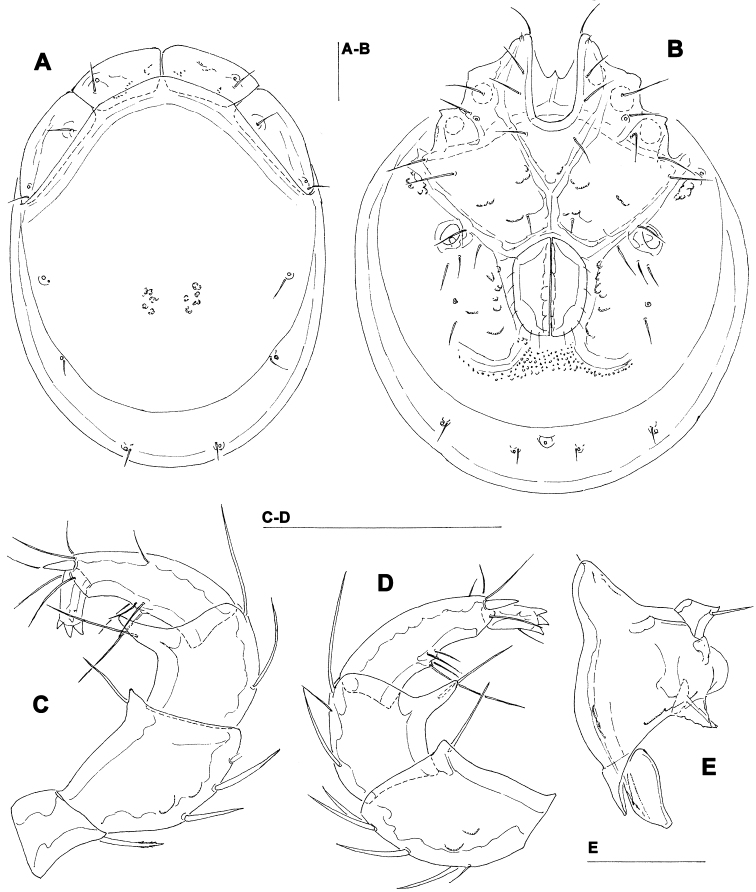
*Torrenticola projectura* sp. n., male: **A** = dorsal shield **B** = idiosoma, ventral view **C** = palp, medial view **D** = palp (P-1 missing), medial view **E** = capitulum. Scale bars = 100 µm.

Male (holotype, in parentheses measurements of paratype, n = 1) — Idiosoma (ventral view: Fig. 4B) L 788 (738), W 675 (625); dorsal shield (Fig. 4A) L 709 (658), W 544 (525), L/W ratio 1.3 (1.25); dorsal plate 663 (614); shoulder plate L 197-200 (189), W 78-82 (71-75), L/W ratio 2.4-2.6 (2.5-2.7); frontal plate L 163 (154-158), W 71-76 (70-72), L/W ratio 2.1-2.3 (2.1-2.3); shoulder/frontal plate L ratio 1.2 (1.2). Capitular bay L 150 (153), Cx-I total L 270 (263), Cx-I mL 119 (109), Cx-II+III mL 80 (70); ratio Cx-I L/Cx-II+III mL 3.4 (3.8); Cx-I mL/Cx-II+III mL 1.5 (1.6). Genital field L/W 156 (159)/124 (128), L/W ratio 1.26 (1.24); ejaculatory complex normal in shape, L 239 (211); distance genital field-excretory pore 180 (150), genital field-caudal idiosoma margin 272 (234). Capitulum (Fig. 4E) ventral L 231 (225); chelicera total L 275 (269), claw L 65 (65), basal segment L 212 (214), L basal segment/claw ratio 3.3 (3.3); palp (Figs 4C-D) total L 257 (256), dL: P-1, 35 (34); P-2, 72 (70); P-3, 52 (53); P-4, 72 (72); P-5, 26 (27); dL P-2/P-4 ratio 1.0 (0.97); P-2 ventrally slightly convex, dorsally convex in the proximal, straight in the distal part, ventrodistal projection cone-shaped, pointed towards distal, P-3 with a long tapering ventral protrusion which curves distally, P-4 distally slightly tapering, slightly curved, ventral setae (one long and three short) on flat hump.

##### Etymology.

The species is named after the distinctive shape of ventrodistal projection of P-3; ‘*projectura*’ - Latinised form of ‘projection’.

##### Remarks.

The shape of ventrodistal projection of P-3 of the new species is very distinctive and can separate it from all other *Torrenticola* species.

##### Distribution.

Taiwan.

### Family Hygrobatidae Koch. Genus *Hygrobates* Koch, 1837

#### Hygrobates (Hygrobates) taiwanicus sp. n.

urn:lsid:zoobank.org:act:8A67823A-EC83-418D-80B8-2934848B1D9D

http://species-id.net/wiki/Hygrobates_taiwanicus

Figs 5 –6

##### Type series.

Holotype female (NMNS-6894-001), dissected and slide mounted, Taiwan, BA-3, Chinkualiao Stream, vi.2010. Paratypes: 0/1/0, same data as holotype; 1/1/0 (mounted), BA-1, Chinkualiao Stream, iii. 2010; 0/3/0, BA-4, Diyu Stream, iii.2010; 0/1/0, BA-5, Diyu Stream, iv.2010.

##### Diagnosis.

Idiosoma L > 750; acetabula arranged in an obtuse angle; female pregenital sclerite with four setae, its anterior margin extending beyond anterior margin of genital plates; female genital plates longer than gonopore; posterior end of female genital plates posterior to postgenital sclerite; P-2 ventrodostal projection elongate, cone-shaped; proximoventral seta on P-4 longer and thicker than distoventral seta.

##### Description.

**Figure 5.**
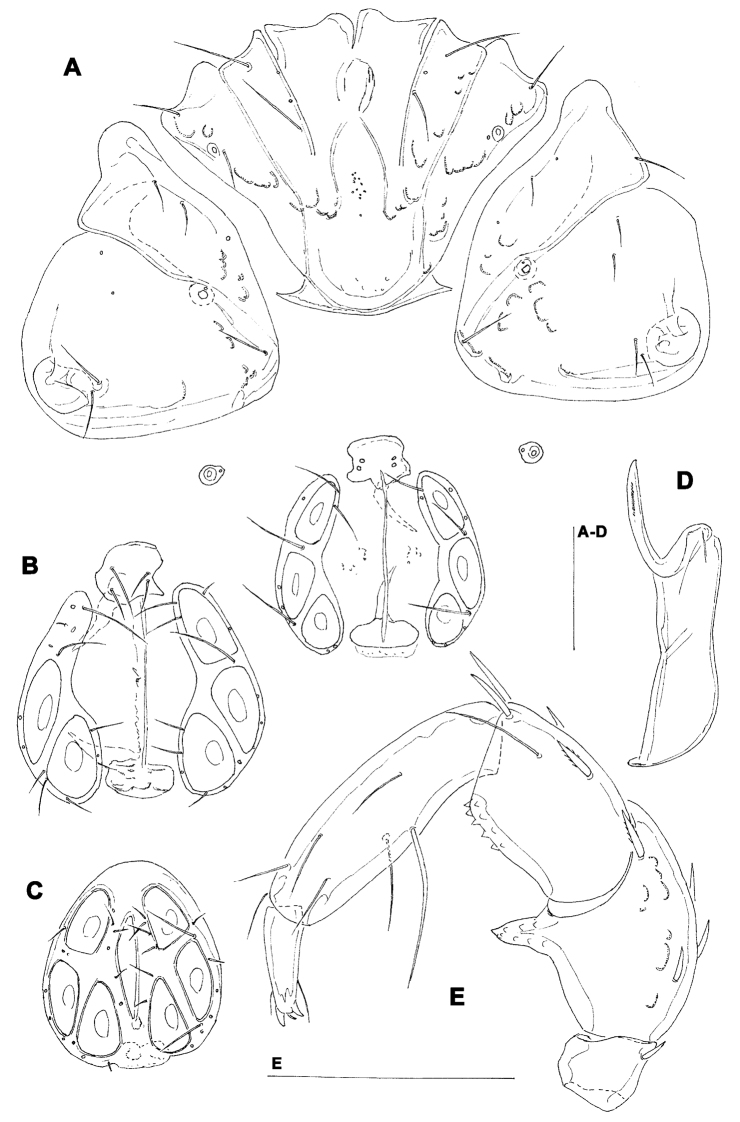
*Hygrobates taiwanicus* sp. n. (**A–B, D–E** = female; **C** = male): **A** = coxal and genital field **B–C** = genital field (paratypes) **D** = chelicera **E** = palp. Scale bars = 100 µm.

**Figure 6.**
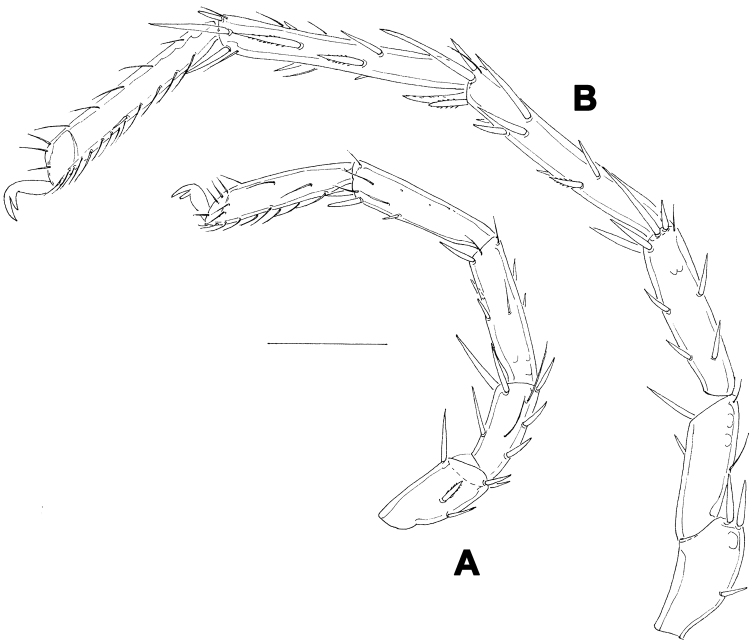
*Hygrobates taiwanicus* sp. n., female: **A** = I-L-2-6 **B** = IV-L. Scale bar = 100 µm.

Female (holotype, in parentheses paratype). Idiosoma L 775 (778), W 613 (706); integument soft, with very fine striation. Capitulum broadly fused with Cx-I. Coxae (Fig. 5A): coxal field L 322 (359), Cx-III W 472 (500), posterior end of anterior coxal group rounded-truncate, Cx-I+II L/W 238 (259)/319 (344), posterolateral apodemes extending slightly beyond sclerotization. Suture line of Cx-III and Cx-IV nearly straight, incomplete, extending to near Cxgl-IV; medial margin of Cx-IV rounded-triangular. Genital field (Fig. 5A-B): L/W 181 (213)/173 (203), acetabular plates with smooth border, L 143-147 (175); acetabula arranged in an obtuse triangle, L Ac-1-3: 55-56 (66), 55-58 (72), 44 (66-68); pregenital sclerite with four setae, its anterior margin extending beyond anterior margin of genital plates. Excretory pore smooth. Palp (Fig. 5E): total L 358 (423), dL: P-1, 26 (34); P-2, 98 (111); P-3, 70 (85); P-4, 121 (136); P-5, 43 (57); dL P-2/P-4 ratio, 0.81 (0.82); chelicera (Fig. FD) total L 250 (319), basal segment L 157 (200), claw L 100 (125), L ratio basal segment/claw 1.6 (1.6). Legs: dL of I-L-2-6 (Fig. 6A): 69 (89), 90 (109), 131 (156), 134 (159), 138 (161); dL of IV-L (Fig. 6B): 108 (128), 103 (128), 151 (184), 213 (263), 225 (272), 193 (234).

Males (n = 2): similar to female, except for genital field. Idiosoma L 775-794, W 625-656; coxal field L 338, Cx-III W 463, Cx-I+II L/W 231/334; genital field L/W 160-170/148-151, anterior margin convex, L Ac-1-3: 51-58, 63-66, 63-66; ejaculatory complex L 169. Palp (Fig. 5C): total L 365-373, dL: P-1, 28; P-2, 99-100; P-3, 71-75; P-4, 119-123; P-5, 48-47; dL P-2/P-4 ratio, 0.81-0.83; chelicera: basal segment L 194, claw L 103, L ratio basal segment/claw 1.9. Legs: dL of I-L-2-6: 74, 91-97, 138-141, 139-141, 142-144; dL of IV-L: 114, 106, 156, 228, 232, 203.

##### Etymology.

The species is named for its occurrence in Taiwan.

##### Remarks.

The new *Hygrobates* species belong to the group of the species characterized byt the presence of two or three setae upon, and inside the margin of, the female pregenital sclerite and proximoventral seta on P-4 longer and thicker than distoventral seta. This group includes three species (Matsumoto et al. 2005), all known only from middle Honshu (Japan), i.e., two lacustrine species, *Hygrobates biwaensis* Tuzovskij, 2003 and *Hygrobates rarus* Tuzovskij, 2003, which are known only from Lake Biwa, and one fluvial species, *Hygrobates capillus* Matsumoto Kajihara & Mawatari, 2005. Due to the elongate distoventral projection of P-2, *Hygrobates taiwanicus* sp. n. is most similar to *Hygrobates biwaensis* (in the following, in parentheses; data taken from Tuzovskij 2003) from which differs in the larger idiosoma dimensions (L 525-650 in both sexes), female genital plates longer (shorter) than gonopore, posterior end of female genital plates posterior (anterior) to postgenital sclerite and P-2 ventrodostal projection cone-shaped (more elongated and slender, finger-shaped).

**Distribution**. Taiwan.

#### Hygrobates (Hygrobates) cf. longiporus

Thor, 1898

http://species-id.net/wiki/Hygrobates_longiporus

Figs 7A–D

##### Material examined.

ECL-BA-3: 23.ix.2009 4/0; ii.2010 0/3/0; vi.2010 2/0. ECL-BA-4: 17.vi.2009 1/0/0. ECL-BA-5: iv.2010 1/0/0; 07.vii.2010 5/0; 27.vii.2010 19/0; 30.x.2010 0/2/0. ECL-BA-6: 17.vi.2009 3/2/0; 17.vi.2009 7/0; vi.2010 0/3/0; 01.vii.2010 2/0/0; 27.vii.2010 12/0 (0/1/0 mounted); 29.ix.2010 0/1/0.

##### Remarks.

**Figure 7.**
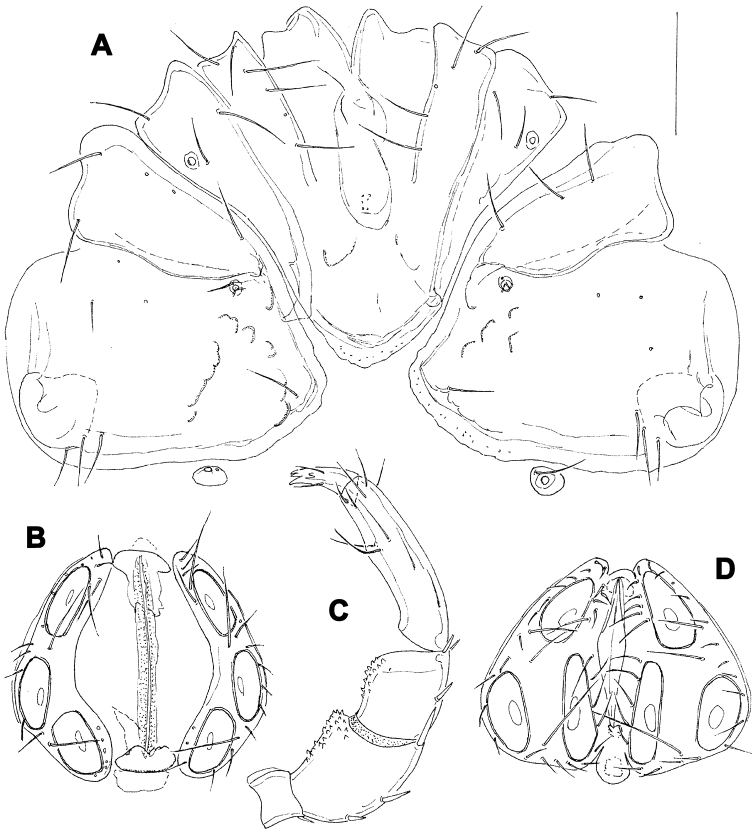
*Hygrobates cf. longiporus* Thor, 1898 (A-C = female, D = male): **A** = coxal field **C** = palp **B, D**  = genital field. Scale bar = 100 µm.

The most abundant *Hygrobates* species from the Baishih River drainage resemble to the Palaeractic *Hygrobates longiporus* Thor. In shape of coxal (Fig. 7A) and genital field (Figs 7B, D) and palp (Fig. 7C), no differences can be found to differentiate the specimens from Taiwan. Two additional species closely related to *Hygrobates longiporus* are described from Oriental part of China (Jin 1997), *Hygrobates neolongiporus* Jin 1997 (Guangxi, Guizhou) and *Hygrobates corimarginatus* Jin 1997 (Yunnan). According to the original description (Jin 1997), *Hygrobates neolongiporus* differs from *Hygrobates longiporus* in Cx-IV medial margins more closely approached to each other, more elongated Ac-3 and the triangular genital sclerites; *Hygrobates corimarginatus* differs in having a fewer denticles on the ventral side of P-2 and P-3 and genital field with more extended border of secondary sclerotization. However, all these characters are probably individually variable, and should be investigated with further specimens from the type area. For the time being, the populations from Taiwan are provisionally assigned to *Hygrobates longiporus*. However, for a judgement on the taxonomic state, the diagnostic features and taxonomic relationship of the Asian species of ‘*longiporus*’ species group require further revision.

##### Distribution.

Palaearctic. New for Taiwan.

#### Hygrobates (Hygrobates) hamatus

K. Viets, 1935

http://species-id.net/wiki/Hygrobates_hamatus

Figs 8A–C

##### Material examined.

ECL-BA-3, vi.2010 0/1/0 (mounted). ECL-BA-5, 20.viii.2009 0/1/0.

##### Remarks.

The specimens from Taiwan are in a good agreement with description of the Oriental *Hygrobates hamatus*. This species is very similar to *Hygrobates soari* K. Viets, 1911, a species widspread in the Afrotropical region, reaching in its distribution to northern Oman (Smit and Pešić 2010). As noted by Pešić et al. (2012) additional material should be studied in order to get an insight into on further diagnostic differences of these two species, what probably will require the application of molecular techniques.

In addition, we gave some measurements of the specimen of *Hygrobates hamatus* from Baishih River drainage which represented the easternmost record finding of this species.

**Figure 8.**
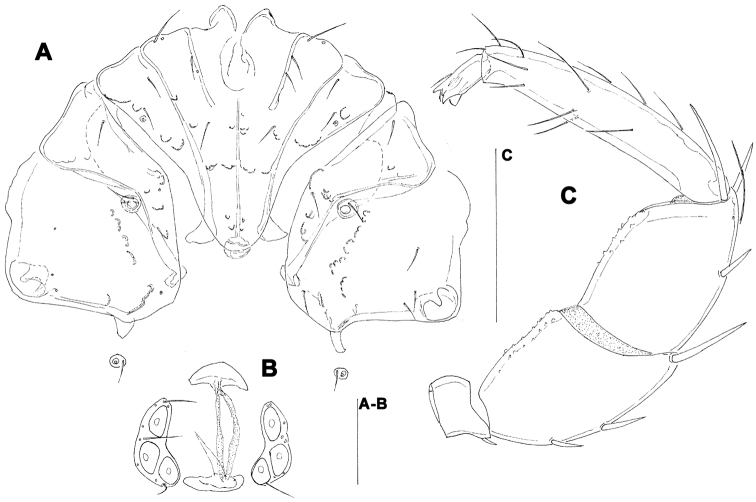
*Hygrobates hamatus* K. Viets, 1935, female: **A** = coxal field **B** = genital field **C** = palp. Scale bars  = 100 µm.

Female. Idiosoma L/W 881/625. Coxae (Fig. 8A): coxal field L 353, Cx-III W 463, Cx-I+II L/W 260/337. Genital field (Fig. 8B): L/W 145/182, acetabular plates L 101-105; L Ac-1-3: 35-37, 38-39, 29-32. Palp (Fig. 8C): total L 442, dL: P-1, 26; P-2, 118; P-3, 101; P-4, 163; P-5, 34; dL P-2/P-4 ratio, 0.72.

##### Distribution.

SE Asia, India, New Guinea, Australia, Iran. New for Taiwan.

### Genus *Atractides* Koch, 1837

#### Atractides cf. spatiosus

(K. Viets, 1935)

http://species-id.net/wiki/Atractides_spatiosus

Figs 9A , 10B

##### Material examined.

ECL-BA-1: 01.vi.2009 0/1/0; 02.vii.2010 0/1/0. ECL-BA-2: iv.2010 0/2/0; vi.2010 0/1/0. ECL-BA-3: 03.ix.2009 0/3/0. ECL-BA-4: iii.2010 0/4/0 (0/1/0 mounted); iii.2010 0/1/0. ECL-BA-5: iv.2010 0/1/0; 07.vii.2010 0/4/0. ECL-BA-6: iii.2010 0/1/0; iv.2010 0/2/0; vi.2010 0/1/0; ix.2010 0/1/0.

##### Remarks.

**Figure 9.**
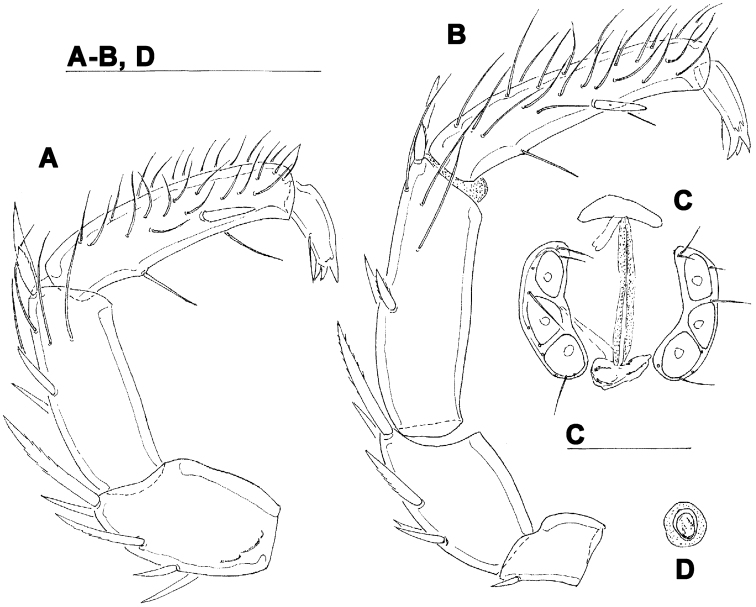
**A** *Atractides cf. spatiosus* (K. Viets, 1935), female: **A** = palp, medial view (P-1 missing) **B–D** *Atractides cf. cognatus* (K. Viets, 1935), female: **B** = palp, medial view **C** = genital field **D** = excretory pore. Scale bars = 100 µm.

**Figure 10.**
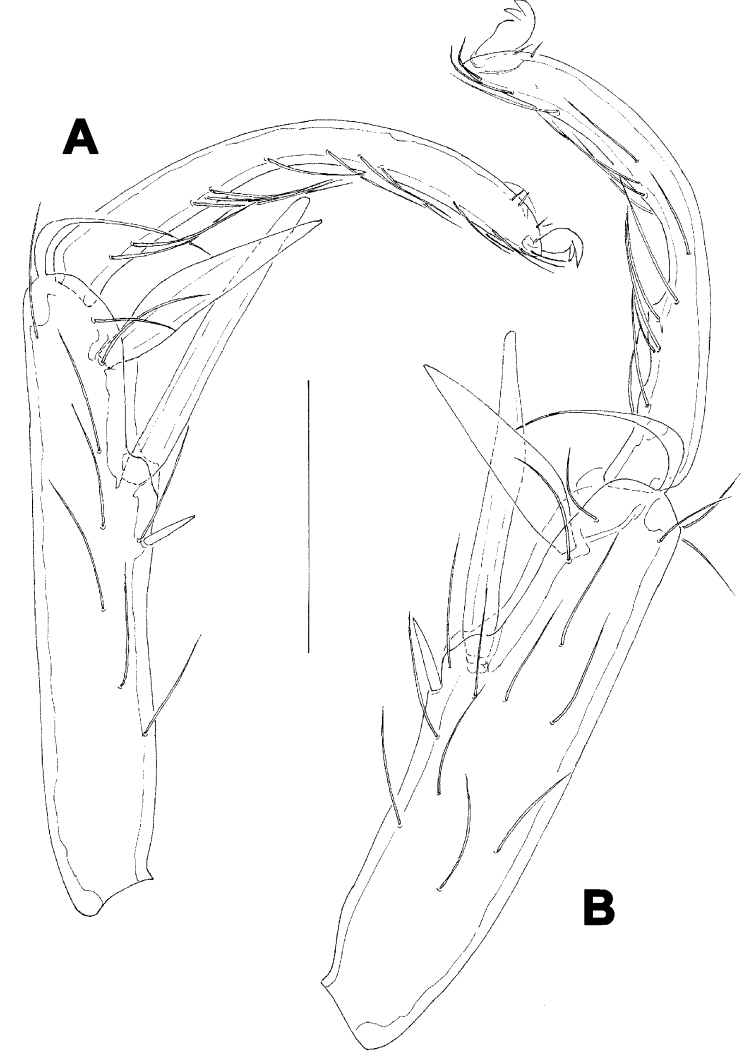
**A** *Atractides cf. spatiosus* (K. Viets, 1935), female: **A** = I-L-5 and -6 **B** *Atractides cf. cognatus* (K. Viets, 1935), female: **B** = I-L-5 and -6. Scale bar = 100 µm.

The examined female specimens from Baishih River system resemble Oriental *Atractides spatiosus* (K. Viets, 1935). A problem exists regarding the similarity between *Atractides spatiosus* and *Atractides cognatus* (K. Viets, 1935), a further taxon originally introduced as a subspecies and later elevated to species rank by Pešić and Smit (2009). However, the populations attributed by Pešić and Smit (2009) to *Atractides cognatus* differ from the original description of the later (see: K. Viets 1935) in the shape of excretory pore (surrounded by narrow sclerotized ring in original description of *Atractides cognatus*), and probably represented an undescribed species. For the time being, the sclerotization of the excretory pore (smooth in *Atractides spatiosus* vs. sclerotized in *Atractides cognatus*, as shown in Fig. 9D), more elongated P-3 (compare Figs 9A and –B) and the shape of I-L-5 and -6 (compare Figs 10A and –B) appear to be the characteristics most important for distinguishing populations attributed to *Atractides spatiosus* and *Atractides cognatus*, respectively. However, our assignments to *Atractides spatiosus* and *Atractides cognatus* are difficult due to the lacking of males, and furthermore is based mainly on non-identity with alternative species.

##### Distribution.

SE Asia. New for Taiwan.

#### Atractides cf. cognatus

(K. Viets, 1935)

http://species-id.net/wiki/Atractides_cognatus

Figs 9B–D , 10A

##### Material examined.

ECL-BA-1: 23.ix.2009 0/1/0. ECL-BA-4: 20.viii.2009 0/1/0; 27.vii.2010 0/2/0. ECL-BA-5: 27.vii.2010 0/3/0; x.2010 0/1/0. ECL-BA-6: 01.vii.2010 0/1/0; 27.vii.2010 0/1/0; 30.x.2010 0/1/0 (mounted).

##### Remarks.

See discussion under *Atractides cf. spatiosus*.

##### Distribution.

SE Asia. New for Taiwan.

## Supplementary Material

XML Treatment for Hydrodroma cf. rheophila

XML Treatment for Sperchon (Hispidosperchon) rostratus

XML Treatment for Sperchon (Hispidosperchon) cf. gracilipalpis

XML Treatment for Sperchon cornutoides

XML Treatment for Monatractides cf. circuloides

XML Treatment for Torrenticola taiwanicus

XML Treatment for Torrenticola projectura

XML Treatment for Hygrobates (Hygrobates) taiwanicus

XML Treatment for Hygrobates (Hygrobates) cf. longiporus

XML Treatment for Hygrobates (Hygrobates) hamatus

XML Treatment for Atractides cf. spatiosus

XML Treatment for Atractides cf. cognatus
